# Flipped classroom in neurophysiology: performance analysis of a system focusing on intrinsic students’ motivation

**DOI:** 10.3389/fphys.2023.1308647

**Published:** 2023-12-08

**Authors:** Maria D. Ganfornina, Sergio Diez-Hermano, Diego Sanchez

**Affiliations:** ^1^ Departamento de Bioquímica y Biología Molecular y Fisiología, Universidad de Valladolid, Valladolid, Spain; ^2^ Departamento de Producción Vegetal y Recursos Forestales, Universidad de Valladolid, Valladolid, Spain

**Keywords:** active learning, inverted classroom, physiology education, student perception, student performance, sentiment analysis, semantic analysis, network analysis

## Abstract

**Introduction:** Teaching methodologies promoting active learning result in higher-order knowledge application, a desirable outcome in health disciplines like Physiology. Flipped-classroom (FC) promotes active learning and engagement in the classroom. Although specialized research keeps accumulating, the advantages of FC for improving academic outcome and ultimately patient care remain controversial and open to further analysis.

**Objective:** This study evaluates the benefits of applying FC to the Neurophysiology module of a Human Physiology course.

**Methods:**We compare final grades of students exposed to standard lecturing (five-years) vs. FC (six-years), and study the FC impact on student motivation, study time and rewards. Differing from conventional FC, we performed no pre-class/in-class assessments, relying on the students’ internal motivation to experience our FC model. A printed student workbook was designed as pre-class material for each session. Reading times respect the expected daily study time of students in our system.

**Results and discussion:** Concerning academic performance, our long-term study reports a significant increase in average scores for FC groups. Overall, students get better scores in multiple choice tests than in problem-solving questions. A more detailed analysis uncovers that our FC model helps students to obtain better scores, reducing variability in performance due to assessment methods. Based on our open-ended survey questions, most students rate the FC environment and in-class activities positively and perceive a positive effect of FC on teachers’ performance. An objective automatic Sentiment analysis of open-ended answers reveals that FC is positively appreciated by students, associating positive perceptions to their understanding of physiological concepts, and negative evaluations to their time management.

## 1 Introduction

The effects of successfully implementing an interactive environment able to promote a self-motivated participation and active learning by students has been amply discussed and reported in many disciplines including higher education Physiology classes (Goodman et al., 2018; Kurtz et al., 2019). However, the highly dynamic nature of the teaching and learning processes is always dependent on the idiosyncrasy of the teacher and student cohorts, which deserves a careful and in-depth analysis by the interested educator.

In this work we report our experience with adopting the flipped-classroom (FC) strategy, a well-known paradigm of active learning (Kaur et al., 2022; Lu et al., 2023), in a total of twelve 1-h lectures comprising the theoretical corpus of the Neurophysiology module within Human Physiology, a mandatory course for second year Medical School students.

Our working hypothesis is that the FC system has a positive effect on learning Neurophysiology. Our project objectives are to investigate: i) the effect of FC on academic performance by comparing evaluation scores of students exposed to standard lecturing vs. FC, and ii) the effect of FC on motivation, study time and reward as perceived by students.

## 2 Methods

### 2.1 Study participants

The undergraduates involved in this study belong to eleven cohorts (during the period 2011–2023), each comprising two groups of ∼90 students (average 180 students/year). In terms of demography, the students show an average 70/30% female/male ratio. Two Physiology teachers (the authors M.D.G & D.S.), with over 20 years of experience in lecturing and teaching Physiology, prepared the pre-class and in-class materials and activities and graded the students in all cohorts. Our aim is to compare 5 years (891 students) instructed with standard lectures and 6 years (962 students) where we used the FC model. The 2020 class was excluded from this analysis due to its singular teaching condition imposed by the COVID-19 pandemics. Attendance to class is not individually registered or rewarded.

### 2.2 Learning subject, pre-class reading materials and in-class activities

Neurophysiology is the last module in our Human Physiology course curriculum. Overall, the subject is perceived as very difficult by students, because of the complexity of anatomic regions, cell types, neuronal circuits, and their underlying membrane biophysical properties.

FC systems generally use pre-class technology-based materials, like slide presentations or educational videos, combined with graded assessments (either pre-class or in-class) of the knowledge acquired before each classroom session. Because of extensive feedback interviews with our students in preceding years about their preference for study materials in paper (that they can read and work with), we decided to go “low-tech” for this project. We published a printed student workbook and study guide (Montaña Díaz et al., 2023) (*Guía de Estudio de Neurofisiología*, GEN) generated through a collaborative work of teachers and a group of medical school undergraduates (former 2nd year students) interested in Physiology education. GEN was based on general Neurophysiology textbooks, the contents of our previous standard lectures, and the students notes, and was thoroughly discussed to select the content (depth and length) appropriate for daily pre-class material. GEN contains 12 chapters, each covering the basic/fundamental concepts of a program topic and designed to be read in 30–60 min. This timing, together with expected after-class work, fits the expected independent study time for the Neurophysiology module according to the European Credit Transfer System (ECTS). GEN also provides illustrations, tables, open questions to reflect on the concepts learned, as well as quizzes for student self-assessment that can be later discussed in tutorial sessions with teachers. Each chapter ends with two-three empty pages for notetaking during class. A pre-class teacher-student communication to solve doubts is available online through our online learning management system (virtual campus). With the purpose of fostering internal motivation to learn and to attend classes, no pre-class assessments are performed. Gaining grade points is therefore excluded as a motivation for adherence to our FC system.

In-class activities aim at promoting theoretical content application, and include: i) doubts-solving; ii) class discussions to identify basic (fundamental) concepts; iii) debates on important topics using the think-pair-share paradigm; iv) short lecturing by teachers and students to explain difficult concepts; v) brief 3–5 min presentations of clinical connections related to Neurophysiology concepts, given by students or teachers using slide or video material; vi) end-of-class challenge questions (“star questions”) to be researched after class by interested students and shared with the community via our virtual campus; and vii) a wrap-up take-home message identified by students or delivered by teachers to underscore two-three large-scale concepts covered in the class. Most activities are used flexibly as required by perceived needs, always available to teachers but distributed in time according to the class dynamics. Examples of these in-class activities can be found in Supplementary File Section S1.

### 2.3 Collection of input variables

A formal assessment of students’ knowledge was carried out through their final examination grade points, derived from a multiple (5)-choice test and open-ended essay questions. An average of 160 students take this exam each year. We evaluate understanding of concepts selected from the pre-class reading material, and their applications to practical cases. Examples of the two types of exam questions are shown in Supplementary File Sections S2, S3.

At the end of the Neurophysiology module (in the last laboratory class), the students filled (voluntarily, without incentives) an anonymous 14-item *ad hoc* questionnaire to appraise their perceptions on learning growth and motivation. The survey covered three topics: i) attendance, materials, and study time; ii) in-class activities; iii) perceptions and expectations. The questionnaire comprised multiple-choice tests, 6-points Likert scale rated assertions, and a free-text suggestions box to share ideas on improving their education and the learning environment. Our survey of students’ perceptions was approved by our university to comply with the General Data Protection Regulation of the European Union.

The number of students participating in the survey ranges 60–170/year, representing an average of 71% of those being graded in the final examination.

### 2.4 Analysis of open-ended survey questions on FC and teachers’ performance

To assess the students’ opinion on the teachers’ skills and whether these perceptions change when we switched to the FC system, we gathered answers from our institutional feedback survey on the teacher’s abilities that favor their learning. These surveys cover the years 2015-2016 (standard lectures; *n* = 30) and 2017–2018 (FC model; *n* = 39). We then asked an independent observer to rate the students’ views using a 5-point scale, with 1 corresponding to an overall negative opinion and 5 to a global positive appreciation.

For a second objective analyzing the students’ perception on the FC system, we classified the students’ comments (a total of 89 paragraphs) into three categories: negative, neutral, and positive. To do so, we performed an automatic Sentiment analysis by means of BART (Lewis et al., 2019), a machine learning model pretrained on millions of Facebook posts. BART is a transformer encoder-decoder (seq2seq) model with a bidirectional (BERT-like) encoder and an autoregressive (GPT-like) decoder. BART is particularly effective for comprehension tasks such as text classification (Li et al., 2022).

After classification, each comment was split into sentences and tokenized into words for network analysis. Common words were discarded. Comments were classified in its original language (Spanish) and translated to English with DeepL for tokenization. Co-ocurrence of words in the same sentence was calculated by computing the inner product of the matrix of word counts. The word “classes” was removed from the analysis as its predominance in all comments prevented the appreciation of other patterns. Networks’ connectivity was measured by means of betweenness and degree. Betweenness index was estimated as $\sum g_{ivj}/g_{ij}$ , where $g_{ij}$ is the total number of shortest paths between vertices i and j , while $g_{ivj}$ is the number of those shortest paths which pass through vertex v . Degree of nodes was estimated as the number of adjacent edges.

All analyses were performed in R programming language (R Foundation, 2023) using the following packages: *transforEmotion* (Christensen and Golino, 2022) for transformer model and classification, *deeplr* (Zumbach and Bauer, 2021) for translation, *tokenizers* (Mullen et al., 2018) and *stopwords* (Benoit et al., 2021) for tokenization and *igraph* (Csárdi and Nepusz, 2006) for network visualization and connectivity measures.

### 2.5 Statistical analysis

The study analysis was performed by a qualified data analyst (the author S.D-H.), blind to the teaching process and input variables retrieval. Statistical analysis was performed in R programming language (R Foundation, 2023). A *p*-value <0.05 was used as a threshold for significant changes. The tests used for each experiment are stated in figure legends. Details of each statistical comparison are listed in Supplementary File Section S4.

## 3 Results

**FIGURE 1:**
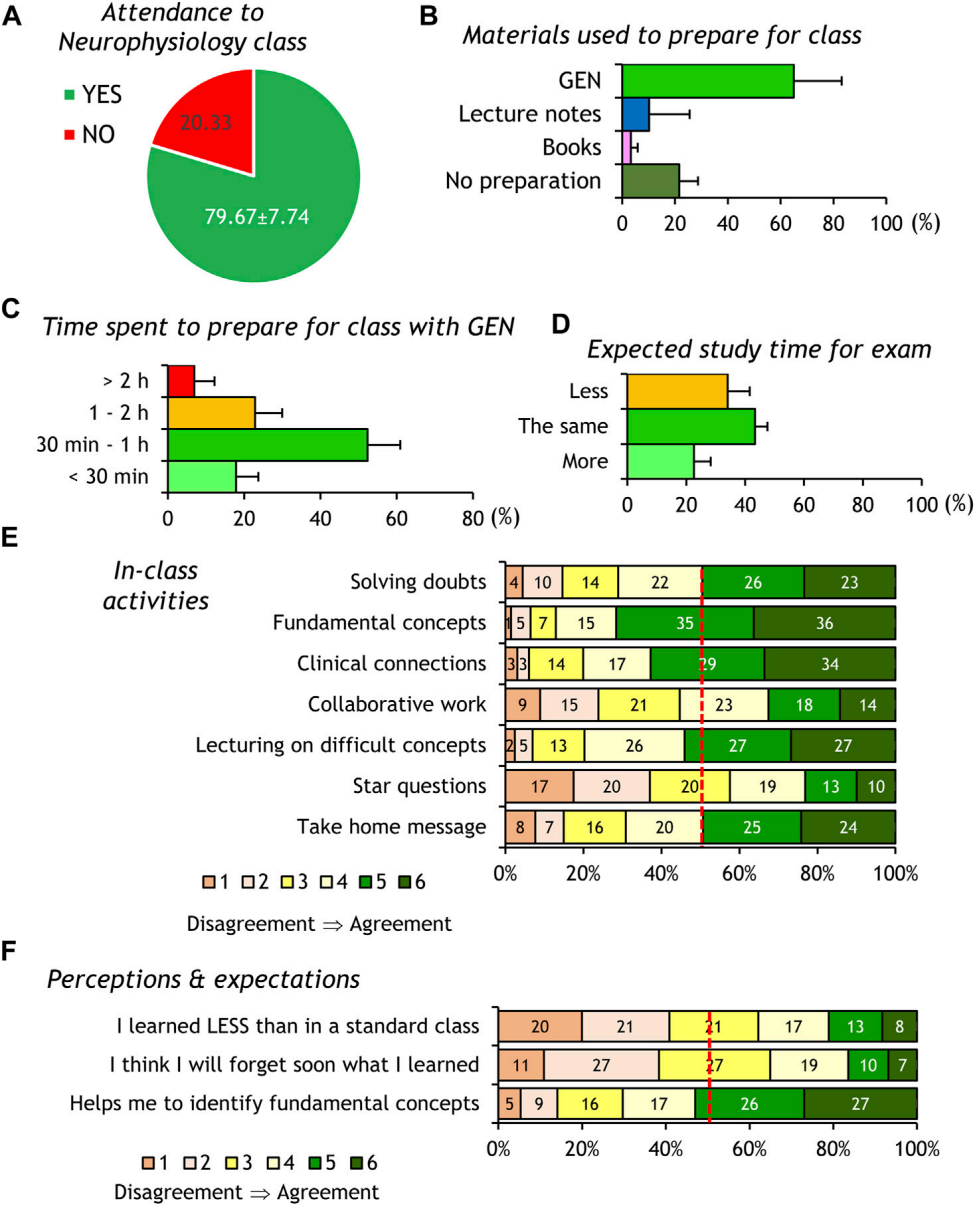
Analysis of survey responses of Neurophysiology students. The panels show average ±SD percent answers to questions concerning class attendance **(A)**, pre-class materials **(B)**, time spent for class preparation **(C)** or expected for exam preparation **(D)**. **(E,F)** The stacked bars show the percentage of students selecting each of the six scale levels for questions exploring the usefulness of in-class activities **(E)** and perceptions/expectations of the FC effects on their learning **(F)**. The dashed red lines in graphs E and F mark the 50% simple majority threshold to visually compare the percent students selecting “disagreement (1-2)” or “agreement (5-6)” in the scale.

The survey to study the effect of FC on students was performed in the last laboratory class of the Neurophysiology module. This questionnaire helped us to estimate an average ∼80% attendance to our Neurophysiology FC sessions (Figure 1A), a necessary estimation since our university policy does not support calling the register in lectures.

Regarding materials used to prepare the forthcoming class, most students used our workbook GEN, followed by study notes from former Neurophysiology students. Few students used books, and ∼20% of the survey respondents did not prepare for the class in advance (Figure 1B). The responses of students using GEN verify the expected 30–60 min average of reading time for the pre-class preparation (Figure 1C).

### 3.1 Perceptions and expectations of FC environment and activities

The question exploring their prediction about additional study time before the final examination (taking place 2-3 weeks after the Neurophysiology module was completed) indicates that students do not consider that the FC paradigm will help them to significantly reduce their time of study, and ∼20% students predict that they will need more time than when receiving standard lectures (Figure 1D).

When asked about the usefulness of the in-class activities of our FC sessions (Figure 1E), most students positively rated (options 5-6 chosen by ≥ 50% of students), those related to solving doubts, highlighting basic concepts, short lecturing, discussing clinical connections and building take-home messages. Only two activities, the “star questions” (perceived as expansions of the topic) and those involving collaborative work (think-pair-share activities), were rated of low interest (options 5-6 chosen by <50% of students).

Most students confirm that FC helps them to identify and learn fundamental concepts (options 5-6 chosen by ≥ 50% of students), but they do not show certainty on whether their FC-derived learning is more efficient or long-lasting (Figure 1F).

### 3.2 Influence of class attendance on student perceptions and expectations

**FIGURE 2:**
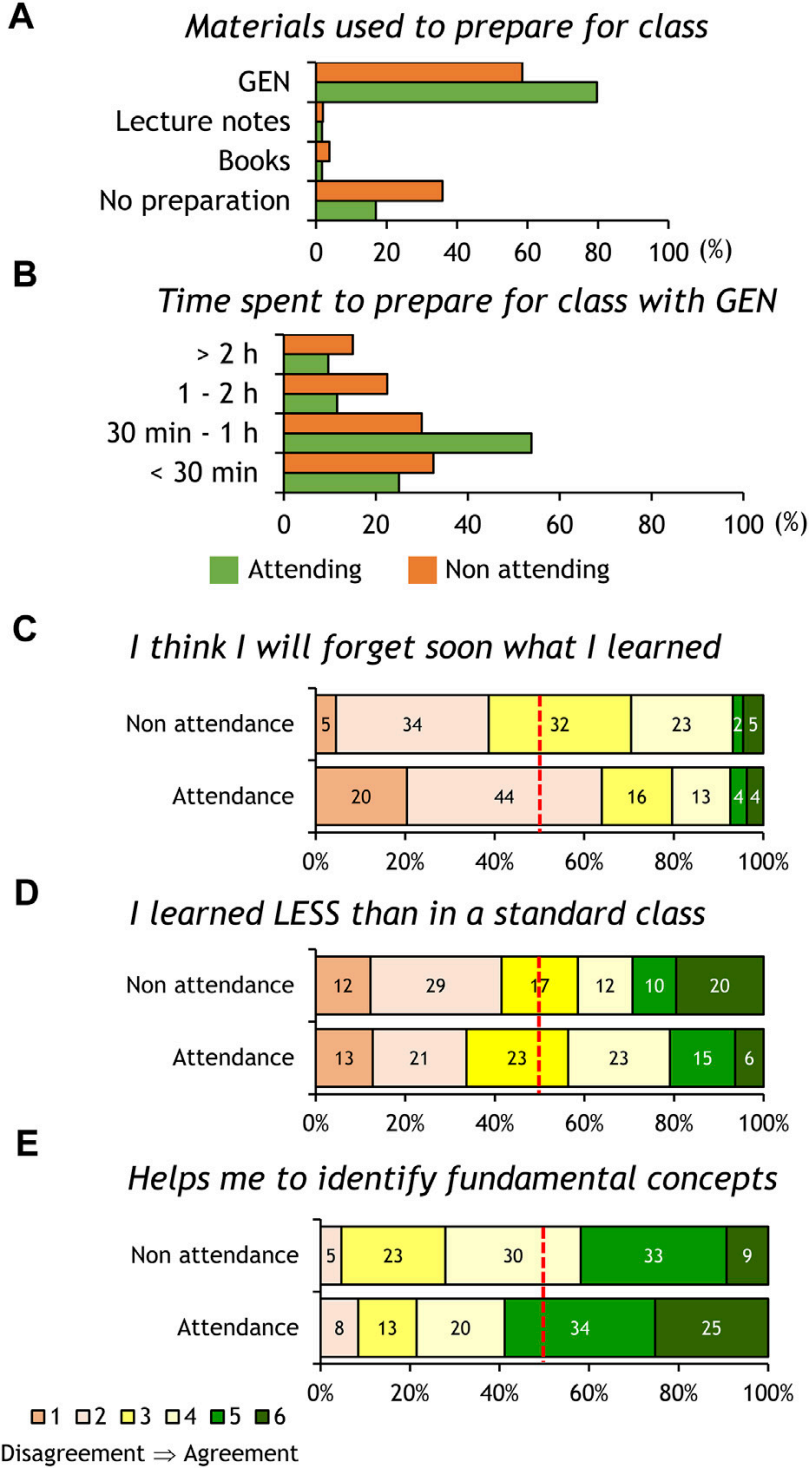
Analysis of survey responses of Neurophysiology students of school year 2022-23 separated into two groups according to their stated class attendance. The panels show answers to questions concerning pre-class materials **(A)**, time spent for class preparation **(B)**, and the frequency distribution of perceptions/expectations of the FC effects on their learning **(C–E)**. The dashed red lines in graphs C-E mark the 50% simple majority threshold to visually compare the percent students selecting “disagreement (1-2)” or “agreement (5-6)” in the scale. Statistical differences were assessed with the Chi-squared test.

Since our anonymous survey was carried out with a clicker system, we were not able to compare the answers of students that regularly attended the lectures to those that do not generally attend. Therefore, in the cohort of the 2022-23 school year we instead used a printed anonymous questionnaire that allowed us to separate both sets of students. Representing these data, we can see that students not attending the FC sessions usually do not prepare for class, and if they do, they spend more time reading the pre-class material (Figures 2A, B).

In relation to perceptions about the benefits of FC, their opinion about forgetting soon the Neurophysiology concepts (Figure 2C) is significantly different between both groups of students (*p* = 0.009), with 63% of attending students showing disagreement (options 1-2) compared to only 38% of non-attending students. The students’ opinion about learning less with the FC model (Figure 2D) does not show significant differences between both groups (*p* = 0.18), but a higher percentage of non-attending students agree with the statement (options 5-6). Both questions suggest that students not attending our FC classes tend to express negative perceptions about FC-based learning. In contrast, both groups of students agree that FC helps them to identify fundamental Neurophysiology concepts (large proportion of options 5-6 marked in Figure 2E). Their agreement shows overall significant differences (*p* = 0.048) with attending students showing a more positive rate (options 5-6 chosen by ≥ 50% of students).

### 3.3 Students’ views of the FC system and teachers by open-ended survey questions

**FIGURE 3:**
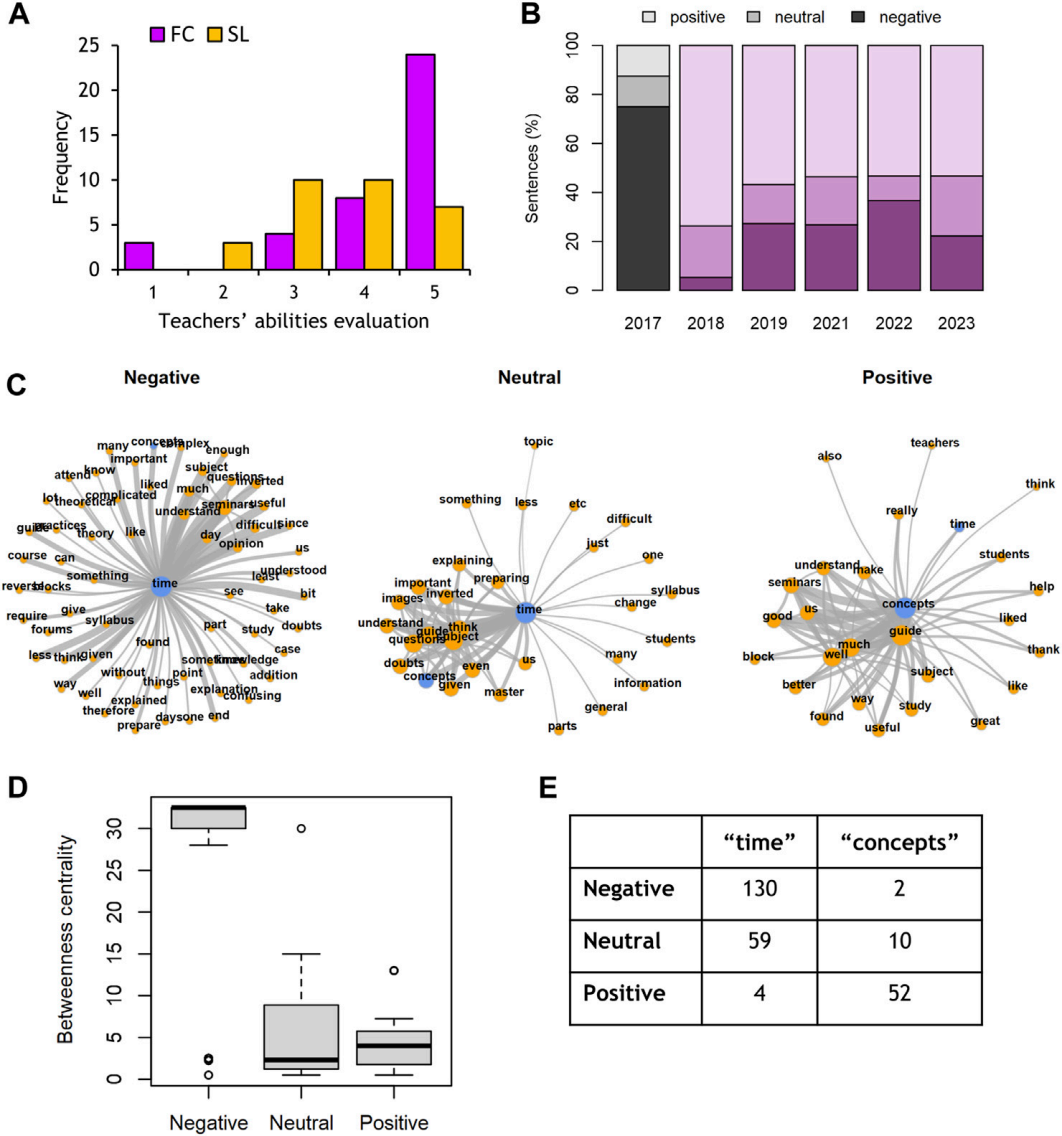
Text analyses of students’ opinions. **(A)** Evaluation of teachers’ performance. **(B)** Sentiment analysis of students’ evaluations of FC environment. Proportion of comments classified as “negative,” “neutral” and “positive” per year (dark to light color code). **(C)** Networks of co-occurrence for the most common words by comments’ sentiment. Only the top 150 co-occurrences are shown. Orange nodes represent words. The terms “time” and “concepts” are highlighted in blue. Edges are depicted by grey curved lines. Two words joined by an edge tend to appear together in students’ comments. Edges’ width indicates frequency of two words co-occurring. Node size represents the degree of the word, which is the number of edges connecting to that particular node. **(D)** Betweenness centrality measure by comments’ sentiment. This measurement is estimated for every node in a network and is a proxy for networks’ centrality. Higher values indicate dominance of a central node, whereas lower values indicate a distributed structure of the network. **(E)** Degree of “time” and “concepts” words. The degree of a node is estimated as the number of edges adjacent to it. The higher the value, the more connections the node has.

Turning our study focus to the teachers’ role in the learning process, we used their responses to open-ended survey questions. We compared the students’ opinions about the teachers’ performance in 2 years of either standard lectures or FC. Plotting the frequencies of the students’ rates (see Methods Section 2.4) demonstrates a clear shift to positive appreciations of the teachers’ abilities after implementing the FC system (Figure 3A), which results in a significant statistical difference when comparing the two groups (*p* = 0.004; Mann-Whitney Rank Sum Test).

Finally, we used Sentiment analysis to assess the students’ evaluations of the FC learning environment. Students’ responses were automatically classified into “negative,” “neutral” or “positive” according to a pretrained machine learning algorithm (see Methods Section 2.4). Expectations were mostly negative for students that had not experienced FC before (Figure 3B, Year 2017). Negative sentiments decreased and stabilized year-per-year around the 20%–30% mark for cohorts that received FC sessions, and positive sentiments rose to 50%–60% (Figure 3B, Years 2018–2023).

Network analysis of most common words revealed differences in the structure of evaluations depending on the sentiment of the responses (Figure 3C). Networks of negative comments were centered around a single node (the word “time”) and had little connectivity, whereas networks of neutral and positive comments had more complex subnetworks and relationships between nodes. This can be measured through the “betweenness centrality” index, which indicates how much a node influences the flow of information in the network (Figure 3D). There was also a gradual shift in the networks’ central term from “time” in negative comments to “concepts” in positive ones (Figure 3E).

Thus, Sentiment analysis revealed that perception of FC learning environment changes after being exposed to it. Negative evaluations seem to orbit around time management, whereas positive perceptions give more weight to the impact of FC on understanding of concepts, with neutral comments being a middle ground, sharing the importance of time with negative views but closer to positive ones in complexity and structure.

### 3.4 FC effect on academic outcomes

**FIGURE 4:**
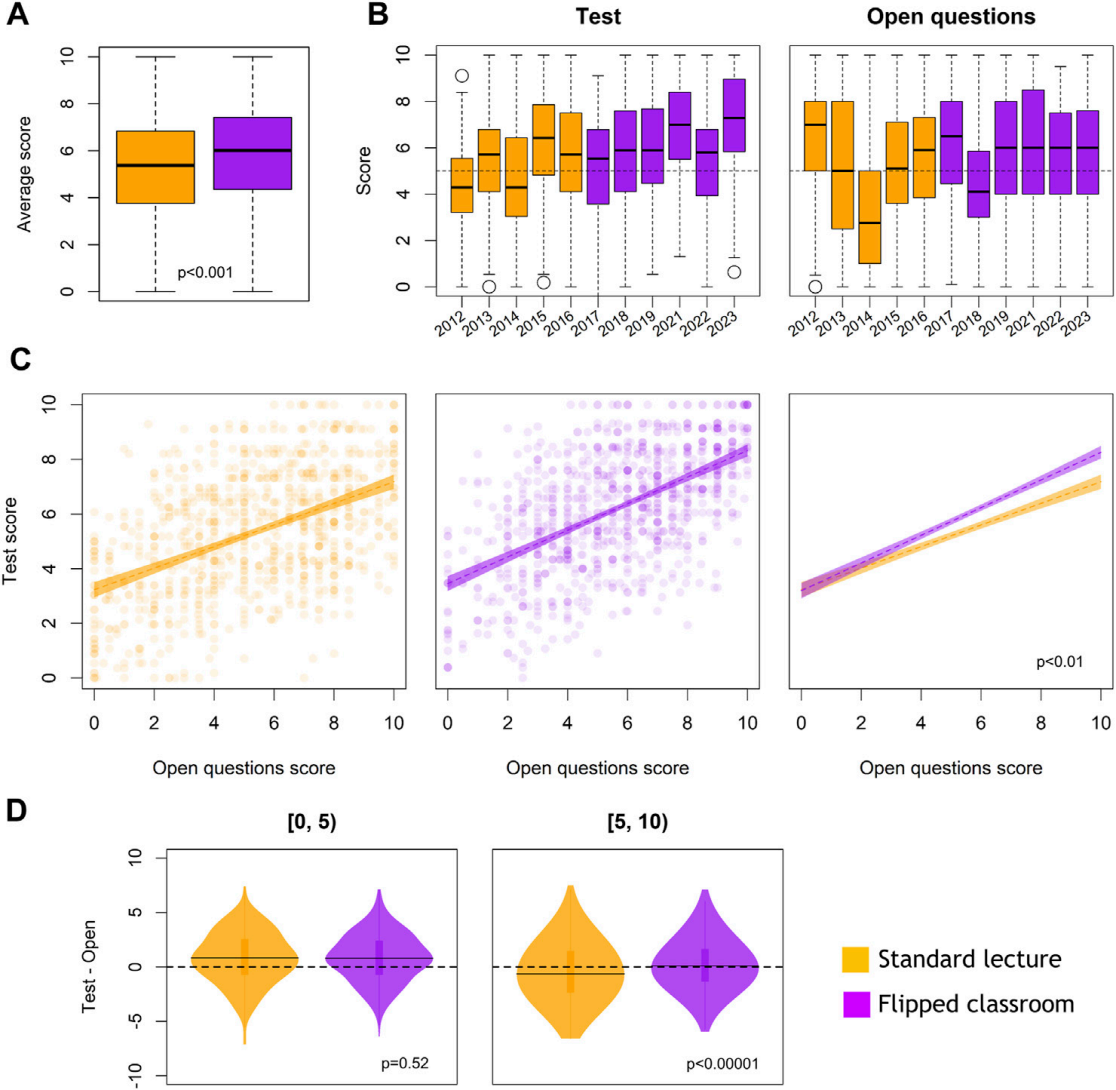
Analysis of academic outcomes in student cohorts with standard lecture (SL) or FC methods. **(A)** Distribution of average scores **(B)** Scores obtained in the multiple-choice test section or in the open question (problem-solving) during the academic years under study. **(C)** Analysis of test vs. open question scores correlations in the SL and FC cohorts. correlations have a different slope, being larger for the FC cohorts. Statistical differences assessed with the Student’s *t*-test. **(D)** Analysis of the variable Test-Open question in the global score ranges 0–5 and 5–10 for the SL and FC groups. Statistical differences were assessed with the Wilcoxon rank test.

We first compared the average scores of test and essay/problem solving questions of the two student samples: courses 2011–16 (standard lectures) vs. 2017–23 (FC). This analysis results in a small but significant better performance for the FC group (*p* < 0.001; Wilcoxon Rank Test) (Figure 4A). We then analyzed the scores obtained in each examination section, (Figure 4B). In general, students get better results in the test than in open-ended problem-solving questions. As expected, test and open questions scores are positively correlated, independently of the teaching methodology used (Figure 4C). However, the slope of the FC correlation does differ from that obtained in the standard lecturing cohorts. This indicates a positive influence of the FC teaching model, but only evident in students that pass the exam (score ≥ 5). This is better visualized in the violin plots of the variable Test-Open analyzed for the ranges 0–5 (not passing the exam) and 5–10 (passing the exam) (Figure 4D). The median of Test-Open score difference is positive in the 0–5 interval, and close to zero or negative in 5–10 score interval. This observation indicates that students that fail the exam have more difficulties in open questions than in the test, independently of the teaching model used. However, the FC teaching model does influence the scores for passing students, by improving their performance at the multiple-choice test. These students show an increased coherence between the two examinations tasks.

In summary, our analysis of students’ academic performance suggests that the FC model benefits students that pass the exam, particularly helping them to obtain better scores in general, and reducing putative variabilities generated by the assessment method (test vs. open tasks).

## 4 Discussion

It is generally accepted that active learning increases student academic performance in science (Freeman et al., 2014). Teaching paradigms that promote active learning, such as FC, have long been advocated to cope with various drawbacks affecting the traditional educational environment (Naing et al., 2023). This is particularly evident in undergraduate classrooms with large number of students who easily choose to skip classes and concentrate on studying for final examinations that usually maximize evaluation of facts and short-memory retention. Moreover, Physiology has been traditionally viewed as a health education discipline centered on learning basic information that would eventually be transferred to and applied in subsequent pathology-related courses.

Many conflicting reports are available about the effects of FC systems on final examination scores (Chen et al., 2018; Gopalan, 2019; Barranquero-Herbosa et al., 2022; Naing et al., 2023). A prospective randomized controlled study finds FC positive effect on grades (Anderson et al., 2017), and a study in advanced Physiology also finds statistically significant improvements in final grades (Rathner and Schier, 2020). However, a recent meta-analysis finds only slight positive effect of FC on final course scores (Gillette et al., 2018).

Our results, though the study was not designed as prospective randomized and controlled, do support slight positive effects on final grades, both quantitative and qualitative (Figures 4A, D). Also, an increase in test scores is observed. Given that the student cohorts are independent, the tendency to get better scores in the test questions might reflect an important aspect worth of further analysis, i.e., an increased teachers’ effectiveness in the design of test questions, since our FC system helps teachers to focus on relevant Physiology concepts that more students are able to grasp. In addition, one could argue that GEN more basic text would favor test scores. To test that possibility, we compared the SMOG readability index (https://www.textcompare.org/readability/smog-index/) of a given GEN chapter (Physiology of central visual pathways) with that of the same chapter in our recommended neuroscience textbook (Augustine et al., 2024). The GEN index (13.6) was in the same readability range as that of the textbook (18.7).

On the other hand, our analysis detects an increased coherence between performance in the two parts of the exam (test and essay/problem solving questions) in students that pass the exam. This positive effect is particularly relevant for high-score students, that can increase their performance in both types of assessment activities.

In the last year analyzed, we have been able to separate the answers of students regularly attending or not to the FC sessions. The analysis highlights an association of non-attending students with lack of pre-class preparation and *a priori* negative attitudes about the benefits that FC could provide. This result agrees with a reported view that an FC effect on assessment performance works only when students comply with class attendance and adhere to the active learning process (Rathner and Schier, 2020). Although adhesion to the learning process is difficult to quantitate, but worth of further study, the predominant positive comments detected in the Sentiment analysis of year 2018 (Figure 3B) does coincide with the teachers’ appreciation of the students’ commitment in pre-class preparation and in-class participation in that particular year.

Although academic performance is being confronted as a weak justification for FC implementation, students’ as well as teachers’ satisfaction amply supports the use of this system in many disciplines. Both education agents appreciate FC positive effects on the classroom environment, motivation and learning appreciation (Campillo-Ferrer and Miralles-Martínez, 2021). Since adhesion to our FC system is not rewarded with grade points, our experience supports the existence of enough intrinsic motivation in our students’ populations to make FC works, resulting both in quantitative and qualitative improvements in performance. Our Sentiment analysis further supports this positive view of the FC system by students. Curiously, positive comments reveal the importance given to the understanding of Physiology concepts. These positive effects highlight FC as a pedagogical method potentiating the linkage of theoretical concepts to practical patient care (Barranquero-Herbosa et al., 2022) and thus, particularly suitable for Physiology classes in health care curricula.

However, why FC adoption by the biomedical educational community is not more prevalent? Several shortcomings of FC vs. standard lectures are patent in the specialized literature: 1) Students’ complaints about the workload and their lack of time to prepare before class. 2) Instructors’ efforts and heavy load to prepare FC-guided teaching. 3) As a consequence of the former, lack of student preparation results in lack of background knowledge, hindering active participation and interactive learning in class.

Students’ concerns about time management are certainly highlighted in our Sentiment analysis, where comments classified as negative by the trained machine learning model create a simple network centered around the single node “time” (Figure 3C). Several studies also report on student complaints about the pre-class workload of FC (Moffett, 2015; Bouwmeester et al., 2016; 2019). We are aware that teachers usually underestimate the preparation load for pre-class materials (Persky and Hogg, 2017). The design of GEN as single pre-class material took that knowledge into consideration without decreasing the level of content complexity, as indicated by our text-readability comparison. Our predicted reading times for GEN (measured by actual reading and understanding by the undergraduate authors) matched the student self-reported reading time (Figure 1C) and confirmed the expected ECTS-based pre-class commitment. This fact, however, does not preclude the subjective worries of students about their time investment.

Faculty time investment has been estimated to be higher for both getting started and maintaining a FC system (McLaughlin et al., 2014). Our perception is that, once GEN was published, the teacher’s time devoted to updating in-class activities is not substantially higher than that used to update our standard lectures. This perception is supported by comparing our biomedical research bibliometric scores, available in our Google scholar and ORCID plots, that appear unaffected by the switch to FC teaching. We do appreciate that the selection of fundamental concepts within each topic, the effort of abridging them to a 30 min reading time for students, and their use as basic units for the design of in-class activities, have helped us improve and enjoy teaching, without work overload.

Thus, we conclude this study by suggesting that our “low tech” way of implementing the FC methodology achieves slight increments in academic outcomes, similar to those reported for standard FC models, without performing pre or in-class assessments which involve a higher workload for teachers and students. Moreover, our students’ perceptions of the value and positive motivation for learning obtained with FC parallels those already reported for this active learning methodology.

Nonetheless, we acknowledge that there is no “best way” to teach or learn, and many factors contribute to the formal effectiveness of instructors, regardless of the teaching model. Therefore, while attaching to the FC system for our Neurophysiology module, we agree that more objective analyses are needed to evaluate the effects of FC on learning outcomes and academic performance, as well as the factors influencing these results, including the effect on teachers. Our analyses stress the importance of preparing short and effective reading materials and practical activities to be of use in an FC environment. This material also allows FC-reluctant students to follow an independent and autonomous learning path, complemented with tutorial help from the teachers. Each student can find her/his “best way” following their intrinsic motivation.

## Data Availability

The raw data supporting the conclusion of this article will be made available by the authors, without undue reservation.

## Supplementary material

SUPPLEMENTARY FILE SECTION Supplementary file contains examples of in-class activities, of multiple-choice questions and open-ended questions, as well as details of the statistical analyses performed.
