# Supplementary material for: Flipped classroom in neurophysiology: performance analysis of a system focusing on intrinsic students’ motivation
Source: Front Physiol. 2023 Dec 8;14:1308647. doi: 10.3389/fphys.2023.1308647 (PMC10749200; doi:10.3389/fphys.2023.1308647)
Supplement: Supplementary file 1 [file DataSheet1.PDF]

Supplementary Material. File 1.

Flipped Classroom in Neurophysiology: Performance analysis of a system focusing on intrinsic students' motivation.

Maria D. Ganfornina, Sergio Diez-Hermano and Diego Sanchez[#]
*** Correspondence:** D. Sanchez, dsanchez@uva.es

1. Examples of in-class activities

The following are particular examples of in-class activities used in our Flipped Classroom method.

1. **Doubts-solving activity.**
A first slide with the title of the lecture is used to prompt the students to open the class by exposing their doubts. The teacher annotates the doubts and then, either directly or using the modular activities prepared for the class, covers all the doubts. The order of going through doubts does not necessary match the order of the student questions. We prioritize keeping the contents within a logical organization.

2. **Fundamental concepts.**
These concepts are identified by students and/or teacher after class discussions, and are compiled in a single slide throughout the class.
An example of a Fundamental Concepts slide in one of the classes devoted to motor systems can contain the following statements:
    - Functions of local circuits.
    - All movements use local circuits
      (which can also participate in reflex movements or generate patterns)
    - Hierarchical organization of motor systems: Levels.
    - Concept and types of Motor Units.

3. **Collaborative work activity.**
We use the think-pair-share paradigm, because of the parallel and continuous benches available in our lecture halls, to help them debate on important or key topics.
The following is an example that can be used in the class devoted to chemoreception: "Differences and similarities between the transduction mechanisms of olfactory and gustatory stimuli". After presenting this statement, a 3 min interval is set, after which students pairs share their responses with the class.

4. **"Difficult concepts" activity.**
They are short lecturing by teachers (or student volunteers) to explain concepts that generate many doubts. They are composed of 2-3 slides that are included in class and used between other activities. An example is the following: "How are K^+, glutamate, glucose, and astrocytes related?".

5. **Clinical connection activity.**
The following example can be used in a class devoted to auditory system (which follows the study of somatosensory system):
A video is played (https://www.youtube.com/watch?v=YMZeBJJ5JJc) while asking students to search if they see something special in the way Evelyn Glennie plays the xylophone.

After that, either the teacher or a pre-recruited student to whom the information (a small explanatory text and the video) was sent the previous day, explains what is special: Evelyn has a serious hearing problem, but still can play and coordinate with the orchestra thanks to her somatosensory system! She plays without shoes and perceives the floor vibrations.

6. **"Star question" activity.**
   They are end-of-class challenge questions to be researched after class by interested students and shared with the community via our virtual campus.
   An example of star questions can be as follows:
   "Can a tennis player have a neuron in her sensory cortex whose receptive field is her racket?"

7. **"Take-home message".**
   We end each class with a wrap-up/take-home message that the teacher delivers or has been identified by students. An example of a "take-home message" slide is the following:
   - In the transduction of auditory stimuli, it is essential:
     - To maintain a high K^+ compartment
     - The structure of the organ and the arrangement of hair cells
   - The auditory system has the fastest and most sensitive mechano-electrical transduction.

2. Examples of Multiple-Choice Test questions

The following are examples of test questions used to assess the final knowledge acquisition by students. They usually test knowledge and recollection of physiology-related facts, but also require some reasoning and relationships between concepts. Questions of different difficulty levels are included in each exam, with a proportion of 50-60% level 1, 20-30% level 2 and 10-20% level 3.

1. The barriers of the central nervous system:
a) allow the bidirectional passage of substances.
b) prevent the free passage of substances in both CSF production and drainage sites.
c) allow ion concentrations to balance between the blood and the brain extracellular space.
d) are present in all brain regions.
e) are regulated by the amount of blood glucose.
Level 1

2. Encoding the .... of a sensory stimulus may require the recruitment of sensory units. Choose the word you would use to complete the statement.
a) Intensity.
b) Location.
c) Duration.
d) Modality.
e) Dissonance.
Level 1

3. Of the following characteristics of the visual stimulus, which one is first processed in secondary visual cortices?
a) The color of an object.
b) The outline (shape) of an object.
c) The recognition of an object
d) The position in space (depth) of an object.
e) Adaptation to darkness.
Level 3

4. The vestibular system...
a) uses a Na^+-dependent transduction mechanism that differentiates it from the auditory system.
b) does not require the existence of barriers between compartments with fluids.
c) requires the existence of a compartment with a high K^+ concentration.
d) is not altered if endolymphatic pressure changes.
e) has circular and antagonistic receptive fields.
Level 1

5. Regarding sensory receptors that detect temperature changes, which of the following statements is true?
a) The same neuron can report small changes or large (harmful) changes in temperature.
b) In a situation of constant temperature, thermoreceptors do not adapt.
c) Thermoreceptors in the skin are responsible for modifying the reference body temperature when it is advantageous to induce fever.
d) Each thermoreceptor has specific molecular receptors that respond in a specific range of temperatures.
e) They require descending pathways for their function.
Level 2

6. A patient affected by COVID-19 has lost his sense of smell (anosmia) for a few months and has subsequently recovered it. Which of the following properties of the olfactory system contribute to anosmia being transient?
a) Compensation by the gustatory system, which generates complex olfactory perceptions.
b) The regenerative capacity of neural precursors in the hippocampus.
c) The existence of a single transduction mechanism in olfaction.
d) The presence of stem cells in the olfactory epithelium that can generate new glia and neurons.
e) The existence of a lactate transport system in the olfactory epithelium.
Level 2

3. Example of Open-Ended Question

Our open-ended questions are generally presented as short physiological or pathological situations that are used as a context to raise questions. They aim at assessing further cognitive skills like reasoning and problem-solving, as well as knowledge synthesis and organization of ideas. We usually guide students reasoning by specific questions within the problem context. This strategy also makes scoring easier and more objective.

NEUROPHYSIOLOGY PROBLEM

1) Garbiñe is a tennis player that has just scored a point (her victory point!). Thanks to a movement made with her arm and hand that carry the racket. The movement was adequate to return the ball with the right speed and direction, so that her opponent could not reach it.

1.1. Briefly describe the role of proprioceptive receptors (specifically, the Golgi tendon organs and the muscle spindles of Garbiñe’s arm and hand), in the execution of this voluntary movement.

1.2. In what circuits do these sensory receptors participate?

1.3. What role do inhibitory interneurons play in local spinal motor control circuits?

1.4. Do these inhibitory interneurons participate in Garbiñe’s voluntary movement?
Yes ☐ No ☐

1.5. To carry out this voluntary movement, an “order” was generated that descends from the central nervous system to the appropriate spinal cord level. Identify the location of neurons whose activity “gave the order” to the spinal circuits used in the movement.

1.6. When did this neuronal activity start? Take the moment when Garbiñe begins to move her arm as a reference point.

2) During the match, Garbiñe's internal body temperature has increased. It is a case of exercise hyperthermia.

2.1. Briefly explain the differences between hyperthermia due to exercise and a fever caused, for example, by a bacterial infection. You can organize your answer in a table.

2.2. What should Garbiñe do after the game, drink water or take an antipyretic drug? Why?

4. Statistics details

Figure	Comparison	N	Test	p-value
2C	Attending vs. Non-attending	108 vs. 44	Chi-squared = 9.31	0.009
2D	Attending vs. Non-attending	110 vs. 41	Chi-squared = 3.31	0.18
2E	Attending vs. Non-attending	107 vs. 43	Chi-squared = 5.66	0.048
3A	SL vs. FC	30 vs. 39	Mann-Whitney = 358	0.004
4C	SL vs. FC	891 vs. 962	Student’s t = 3.32	0.0009
4D	SL vs. FC [0,5)	382 vs. 327	Wilcoxon = 62567.5	0.51
4D	SL vs. FC [5,10]	509 vs. 635	Wilcoxon = 134529	4.4e-6
